# Novel Formulation of Bigel-Based Vegetable Oil Spreads Enriched with Lingonberry Pomace

**DOI:** 10.3390/foods11152213

**Published:** 2022-07-25

**Authors:** Gintarė Baltuonytė, Viktorija Eisinaitė, Rita Kazernavičiūtė, Rimantė Vinauskienė, Ina Jasutienė, Daiva Leskauskaitė

**Affiliations:** Department of Food Science and Technology, Kaunas University of Technology, Radvilenu pl 19, LT-50254 Kaunas, Lithuania; jukusike@gmail.com (G.B.); rita.kazernaviciute@ktu.lt (R.K.); rimante.vinauskiene@ktu.lt (R.V.); ina.jasutiene@ktu.lt (I.J.); daiva.leskauskaite@ktu.lt (D.L.)

**Keywords:** bigel, spread, food design, lingonberry

## Abstract

In this study, bigel-based vegetable oil spreads with lingonberry pomace addition were prepared. The impact of gelatin, agar and collagen was examined as structuring agents as was the effect of lecithin concentration (0.5, 1.0, 1.5%). Prepared systems were evaluated by physical and chemical stability and structural and rheological properties. It was found that all bigel formulations were self-standing with no signs of phase separation at ambient temperature immediately after preparation and after two weeks of storage at 4 °C temperature. The lingonberry pomace addition affected grainy structure formation with homogenous and uniform distribution of fiber particles throughout the bigel matrix and it also altered the colour of the bigels toward a purple-red. Texture, rheological properties and colour of the spread formulations were affected by the type of the structuring agent as well as the lecithin concentration. The presence of the lingonberry pomace enhanced the resistance of the bigel samples to the oxidation process and it was confirmed by the DPPH^•^ inhibition, peroxide value and oxipress test. Overall, the formulated bigel-based spreads could be beneficial and had a potential application as healthier fat spreads and be a source of dietary fibers (11 g of fibre per 100 g of the spread).

## 1. Introduction

In recent years there is a growing trend towards healthier food products containing lower amounts of the saturated or trans fatty acids. To formulate alternatives with high nutritional quality is still challenging because of the palatability, functionality and texture provided by conventional fat [1,2]. In order to mimic the structural and sensory properties of solid fat, various techniques and materials had already been used. Among all of them an emerging oleogelification technology looks very promising, as semi-solid properties in oils could be obtained without modifying their chemical characteristics [3]. Oleogels were already successfully used as a healthy alternative to fully or partially replace the saturated fat present in chocolate spreads [4], functional chocolate spread [5] or filling creams [6]. Despite the potential of these systems, the application of those are still limited because of their very high fat amount, limited textural properties and incompatibility with some food matrixes [7,8]. It is a reason why we focused on novel complex biphasic systems, also known as bigels. Because of the different polarity phases, these systems have both oleogels and hydrogels advantages. It was previously reported that bigels posses many unique characteristics such as simultaneous encapsulation of hydrophilic and lipophilic components [9], easy preparation [10], good mechanical stability, easily managed system properties and spreadability [11]. Spreadability of the bigels looks to be a very attractive property as it could be applied in the healthier spread formulations. However, bigels were already used as cream analogues [12], as vitamin E, lycopene, β-carotene and a probiotic delivery system [13,14,15] and even as potential food ink for the 3D printing [16]. However, these systems were never applied in the food-grade spreads preparation. Moreover, we noticed that there is a lack of fat-based spreads enriched with fibers. This may be explained by the incompatibility of polarity between the oil phase and fibers. In that case, the hydrogel phase appearance in the bigel composition could serve as a carrier and immobilizer of the fibers in such fat-based spreads.

Lingonberries are generally valued for their high amount of nutrients and various bioactive compounds such as vitamin C, flavonoids, anthocyanins, phenolic acids, flavonols and proanthocyanidins [17,18]. These active substances are well known due to their antioxidative, antibacterial, antiinflammatory activities and hypoglycemic profile [19,20]. If not consumed, fresh lingoberries are mostly processed into juice and jams that result in a high amount of byproduct formation. It was previously reported that lingonberry pomace composed of skins and seeds are rich in bioactive compounds [21]. Moreover, most of the soluble and insoluble dietary fibers during the manufacturing process of berries remain in press residue or pomace. A number of studies related the beneficial health effects in the human body with the fiber consumption [22]. However, despite that, the by-products are rich in high-added value compounds that are rarely re-used in other products formulation.

Thus, the aim of the study is to design bigel-based vegetable oil spreads with the addition of lingonberry pomace and examine the impact of gelatin, agar and collagen as structuring agents and the effect of lecithin concentration. Prepared systems were evaluated by physical and chemical stability and structural and rheological properties.

## 2. Materials and Methods

### 2.1. Materials

For the oil phase (oleogel) preparation a mixture of sunflower oil and olive pomace oil (composition: saturated (10.8 g/100 mL), monounsaturated (32.8 g/100 mL) and polyunsaturated (48.3 g/100 mL) fatty acids) was purchased from the local supermarket (Basso, San Michele di Serino, Italy) and used without further processing. Carnauba wax (melting point 82–84 °C) obtained from Sigma-Aldrich (St. Louis, MO, USA) was used as an oil structuring agent. Collagen (bovine, I and III type mixture; 90% of protein) was obtained from MyProtein (Manchester, UK). Emulsifier soy lecithin and agar was kindly offered by the Alvas Group (Kaunas, Lithuania). Gelatin was obtained from Dr Oether (Kladno, Slovakia). Stevia sweetener was obtained from the Alvo Group (Kaunas, Lithuania).

Lingonberry pomace left after juice pressing was dried at 35–38 °C temperature in the drying chamber (Thermofisher Scientific, Waltham, MA, USA) for 48 h and ground by a ZM 200 mill (Retsch, Haan, Germany) using a 0.5 mm sieve. The composition of the pomace was as follows: moisture–3.41%; ash–1.18%, protein–8.60%; fat–12.68%; carbohydrates–4.43%; total insoluble dietary fiber–65.36%; total soluble dietary fiber–8.49%; total phenolic content-6.26 GAE/g d.m.

### 2.2. Methods

#### 2.2.1. Preparation of Bigel-Based Vegetable Oil Spreads

Different formulations of bigel-based vegetable oil spreads were prepared. Oleogels were prepared by dissolving carnauba wax (10%, *w*/*w*) and different amounts of lecithin (0.5, 1.0 and 1.5%, *w*/*w*) in oil phase under agitation at 85 °C until complete solubilization. Four different types of the hydrogels were prepared: with gelatin (5% *w*/*w*), with a gelatin (5% *w*/*w*) and collagen (15.6% *w*/*w*) mixture, with agar (2.5% *w*/*w*) and with agar (2.5% *w*/*w*) and collagen (15.6% *w*/*w*) mixture. After collagen and/or gelatin were dispersed into the distilled water, mixtures were kept at 85 °C in a water bath for 30 min. Different from other structuring agents, agar was mixed with distilled water and kept for 1 h at room temperature before placing it in the water bath.

Hydrogels were poured into the oleogels at a ratio 40:60, and then cranberry pomace was added and mixtures were homogenized for 2 min at 15,000 rpm at 85 °C. Immediatelly after the homogenization, samples were added to the icy water and kept until full solidification. Later, samples were transfered to the refrigerator and kept for 24 h before the analysis. The composition of the different spreads are presented in Table 1; Table 2.

The regulation (EC) No 1924/2006 states that a claim that a food is high in fibre, and any claim likely to have the same meaning for the consumer, may only be made where the product contains at least 6 g of fibre per 100 g. In our case the amount of the fibers reached ~11 g of fibre per 100 g of the spread.

#### 2.2.2. Texture

Measurements were performed with a TA-XT2 texture analyzer (Stable Micro Systems, TA.XT Plus, Godalming, UK) equipped with a 30 kg load cell. About 100 g bigel was formed in a 100 mL glass beaker (50 mm in diameter and 30 mm height) and penetrated to a distance of 10 mm at a speed 1 mm/s with a 5 mm spherical probe and then returned to a starting point. Force–distance curves were obtained from the penetration tests and hardness as well as cohesiveness were used for the spreads characterisation.

#### 2.2.3. Colour

A Minolta Chroma Meter colorimeter (Model CR-310, Japan) was used to measure the colour of the samples. The results were expressed as L*, a* and b* values and whiteness index (WI). The whiteness index (WI) was calculated from the following Equation (1):

(1) $$\mathrm{WI}=100-{\left[{\left(100-L^{*}\right)}^{2}+a^{*2}+b^{*2}\right]}^{1/2}$$

#### 2.2.4. Rheological Properties

A rotational dynamic shear rheometer (Physica MCR502, Anton Paar, Messtechnik, Stuttgart, Germany) was used to characterize the rheological properties of the bigel samples. A cone-plate geometry (D 25 mm, angle 2°, truncation 99 µm) with 1 mm gap was adopted. Frequency sweeps of the samples were conducted within the linear viscoelastic region at a fixed shear strain (0.05%) to determine the changes in the elastic (G′) and viscous (G″) moduli within the increasing frequency range from 1 to 100 rad/s (temperature 25 °C).

#### 2.2.5. Oil and Water Binding Capacity

The oil and water binding capacity was evaluated after centrifugation of 10 g sample at 5000 rpm for 10 min at 25 °C using a centrifuge MPW-260RH (Med Instruments, Fujan, China). The total amount of liquid released was poured to the glass and weighted and used for the total fluid released (%) calculation according to Equation (2):

(2) $$Total\;fluid\;released\;\left(\%\right)=\frac{Mass\;of\;the\;released\;fluid}{Mass\;of\;the\;sample}\cdot 100$$

The oil and water binding capacities were determined by heating the glass with the fluid at 105 °C in an air thermostate (Thermofisher Scientific, Waltham, MA, USA). The fat released was calculated from the residual weight and the total weight of the fluid. Water release was calculated as the difference between total fluid released and fat released.

#### 2.2.6. Oxidative Stability

The formation of primary oxidation products (hydroxyperoxides) was measured under accelerated conditions at 120 °C (0.5 MPa pressure) using Oxipres apparatus (Mikrolab Aarhus, Højbjerg, Denmark). In the Oxipres apparatus, the oxidation process is assessed by the rapid increase of oxygen consumption by the oil, which is registered as a pressure drop in a hermetically sealed vessel pressurized with oxygen. The sample weight was 15 g, and the induction periods (IPs) were determined. Each sample was measured in duplicate and the results presented as mean values. For measuring peroxide values the bigel samples were kept at 37 °C for 30 days to accelerate lipid oxidation. Peroxide values were determined according to the LST EN ISO standard (ISO 3960:2017) titration method.

#### 2.2.7. Antioxidant Capacity

For the antioxidant capacity measurement, 2 mL of DPPH^•^ (6 × 10^−5^ M) solution was mixed with 50 µL supernatant solution in a 1 cm path length disposable microcuvette (Greiner Labortech, AR Alphen aan den Rijn, The Netherlands) and the changes in the absorbance of the reaction mixture were measured after 40 min at 515 nm. Absorbances were measured with a Spectronic Genesys 8 spectrophotometer (Thermo Spectronic, Rochester, NY, USA).

The final RSC values were calculated by using a regression equation of Trolox concentration (0.015625–0.25 mg/mL). The antioxidant capacity of each sample is expressed as mg of Trolox equivalent (TE) per gram of bigel.

#### 2.2.8. Statistical Analysis

All analyses were carried out in triplicate. The results are presented as the mean ± standard deviation. A *p*-value of <0.05 was used to indicate significant differences between the mean values determined by an analysis of variance (ANOVA) using Statistica 12.0 (StatSoft, Inc., Oklahoma, AK, USA, 2013).

## 3. Results and Discussions

### 3.1. Effect of Lecithin Amount on the Properties of Bigel-Based Vegetable Oil Spreads with Lingonberry Pomace, Structured with Gelatin or Gelatin and Collagen Mixture

**Figure 1:**
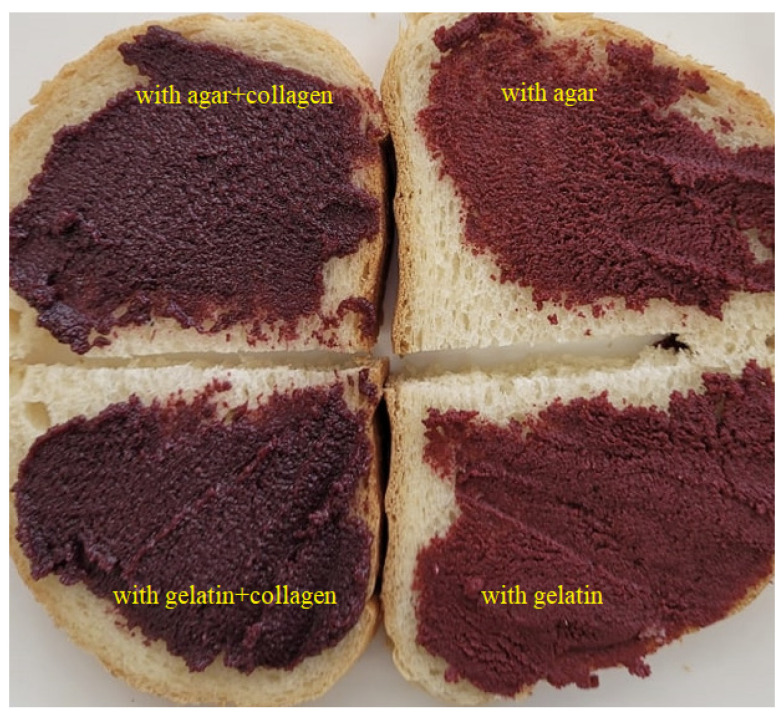
Images of bigel-based vegetable oil spreads structured with different hydrophilic components (0.5% lecithin in oleogel fraction).

All bigel formulations were self-standing with no signs of phase separation at ambient temperature immediately after preparation and after two weeks of storage at 4 °C temperature. Figure 1 shows that all bigels were characterized as easily spreadable, which laid a foundation for their applications as edible spreads. It is also worth to mention that, contrary to our expectations, lecithin concentration did not affect bigel spreadability. The stability and textural properties of the formulated bigels was highly dependent on the self-assembling properties of lipophilic (carnauba wax) and hydrophilic (gelatin and collagen) gelators. Carnauba wax forms crystalline networks in the oil phase by imobilizing liquid oil, thus preventing the mobility of lipophilic compounds [23]. Meanwhile, gelatin is an excellent aqueous gelator responsible for the hydrogel structure formation. The good structuring properties of gelatin were previously reported by [24] in gelatin-based sesame oil/soybean oil emulgels and bigels. It was also found that bigels were easily spreadable, as can be seen in Figure 1.

The addition of Lingonberry pomace affected the grainy structure formation with a homogenous and uniform distribution of fiber particles throughout the bigel matrix, and it also altered the colour of the bigels toward a purple-red (Figure 1). The first clearly seen thing is that spreads made with collagen were more dark and purple, and this was also confirmed by the lower L* values (24.35–27.71) (Table 3). It is most likely that this was affected by the darkish brown color of the hydrolysed collagen solution formed during the bigel preparation. Regarding the effect of lecithin concentration, it was discovered that lower lightness (L*), higher redness (a*) and yelowness (b*) values were found in the samples with the highest lecithin concentration (1.5%). This could have been caused by unequal droplet size distribution resulting in differences in light diffraction [25]. Changes in colour and turbidity in the bigels with κ-carrageenan hydrogel and monoglyceride oleogels were previously explained by the same phenomenon [14].

The presence of a higher lecithin concentration in the bigel formulation led to a lower dynamic stability. TFR values increased from 16.11 to 21.82% in samples with gelatin and from 17.75 to 25.93% in samples with a gelatin/collagen mixture after lecithin concentration increased from 0.5% to 1.5% (Table 3). One of the possible explanations of this result could be hydrophobic or electrostatic interactions between lecithin and proteins occuring on the water-oil interphase [26,27], which are not always desirable and could cause unfavorable changes in protein conformation [28]. This assumption was also confirmed by the fact that higher TFR values were found in samples with higher total protein content (with gelatin and collagen), potentially promoting more interactions. As shown in Table 3, the highest part of the released fluids was oil (98–99% of the total fluid released), most likely due to the ratio of oleogel to hydrogel 60:40.

**Figure 2:**
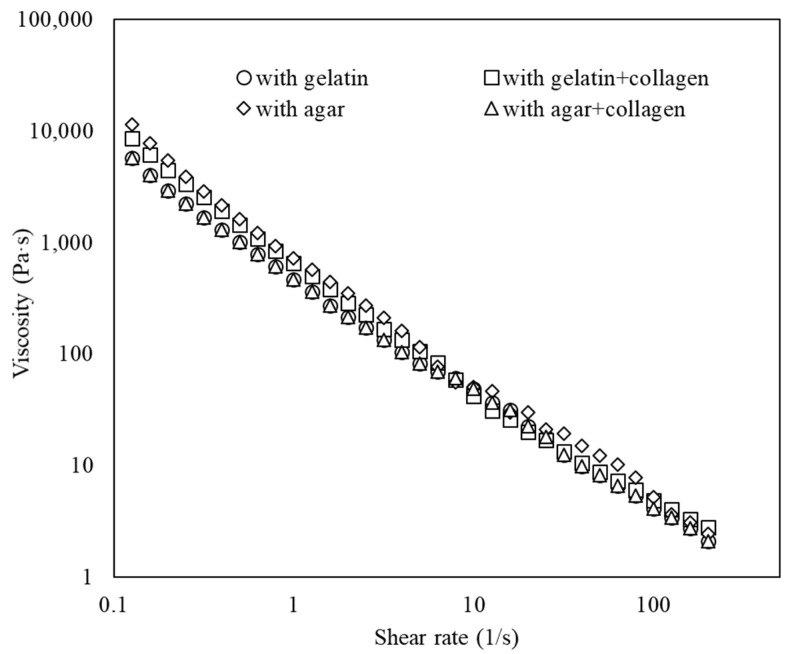
Viscosity-shear rate profile of different bigel-based vegetable oil spreads (with 0.5% lecithin concentration in oleogel phase).

All spread formulations exibited non-Newtonian shear-thinning behaviour with flow index n < 1 and showed great pseudoplasticity (Figure 2). The network of both gel phases were destroyed and exhibited lower viscosity values after a high shear rate was applied. It was previously reported that shear-thinning behavior was also strongly linked with the spreadability of the samples [29]. It also could be seen (Figure 2) that in all samples the viscosity-shear rate profile was the same, meaning that the type of bigels had no correlation with their viscosity.

It was also discovered that G′ was greater than G″ in all bigel formulations over the range of frequencies studied. Therefore, the absence of crossover points between the storage and the loss moduli indicated their predominant solid-like elastic character [30]. That was also confirmed by the loss factor (tan δ) values that were between 0.1 and 1.

**Figure 3:**
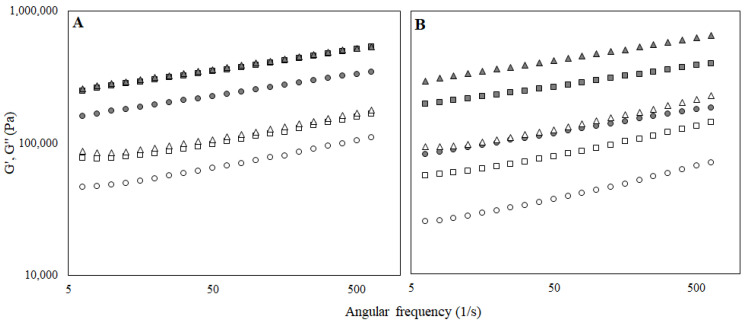
Elastic (G′) and loss modulus (G″) of the bigel-based vegetable oil spreads structured with gelatin (**A**) or gelatin and collagen mixture (**B**) with different concentrations of lecithin (○—0.5%; □—1.0% lecithin; ∆—1.5%) in the oleogel fraction.

However, frequency dependence was observed for all the samples (Figure 3), and according to [31], the dependency of G′ and G″ to the applied frequency indicated the weak gel strength. It is noteworthy that oil and aqueous phases were interpenetrating synergistically and create a semi-continuous network, indicating that pomace addition had no negative effect on the obtained structure. According to Figure 3, G′ and G″ significantly increased by increasing the lecithin content in the bigel sample, indicating a high degree of structuration. Also, it was found that the G′ and G″ values of the bigels structured with gelatin was slightly lower than the corresponding bigels with additional collagen.

It was previously reported that the spreadability of semi-solid materials is inversely proportional to their firmness [30]. With the increase of the lecithin concentration, bigels showed a significant decrease of the firmness, from 5.14 to 2.88 N (with gelatin) and from 3.75 to 2.31 N (with gelatin and collagen). In the samples with additional collagen, lower firmness values correlated with lower G′ values.

**Figure 4:**
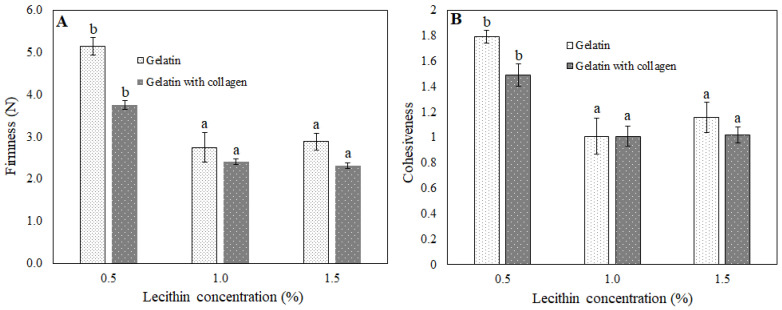
Firmness and cohesiveness of bigel-based vegetable oil spreads structured with gelatin (**A**) or gelatin and collagen mixture (**B**) with different concentrations of lecithin in the oleogel fraction. Data labeled with different letters showed statistically significant differences (*p* < 0.05) between bigel-based vegetable oil spreads formulation with different lecithin concentrations.

One more important textural property is cohesiveness, which indicates the strength of internal bonds and recoverable structure after the first bite [32]. Similarly to hardness, cohesiveness was significantly influenced by lecithin concentration (Figure 4). It also worthy of mention that correlation between hardness and cohesiveness values was observed as more firm samples were more cohesive, which is similar to that found in other studies [33].

### 3.2. Effect of Lecithin Amount on the Properties of Bigel-Based Vegetable Oil Spreads with Lingonberry Pomace, Structured with Agar or Agar and Collagen Mixture

Agar is a well- known natural polysaccharide that has good gelling properties, mechanical strength and sol-gel transition properties similar to gelatin [34]. This is the reason why agar was chosen for the bigel-based vegetable oil spreads preparation as an alternative to gelatin of animal origin. It was previously reported that agar produces firm gels at a concentration of 1% (*w*/*w*) [35]. Thus, this concentration was our choice as sufficient for hydrogel structure formation. There are not many studies about agar inclusion in the bigel composition. It was previously reported that bigels were prepared with gelatin-agar mixture hydrogel, however these systems were used for the drug encapsulation [36].

**Figure 5:**
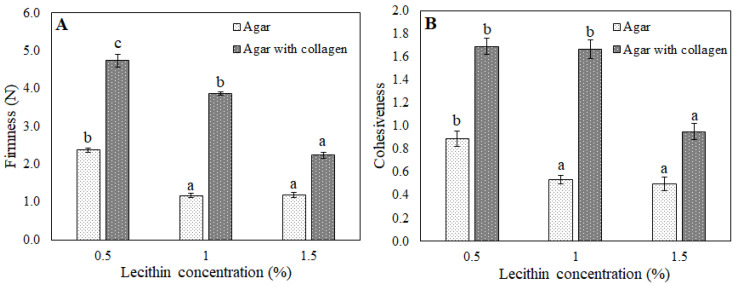
Firmness (**A**) and cohesiveness (**B**) of bigel-based vegetable oil spreads structured with agar or agar and collagen mixture with different concentrations of lecithin in the oleogel fraction. Data labeled with different letters show statistically significant differences (*p* < 0.05) between bigel-based vegetable oil spread formulations with different lecithin concentrations.

As in the case of the gelatin-based spreads, the difference observed between samples could be attributed to the different hydrogel composition (with or without collagen) as well as different lecithin concentrations. Regarding the colour, the systems with collagen had the same tendency as previously described samples: they were darker and more purple (Figure 1; Table 4). However, different from the gelatin structured spreads, the TFR was lower in the systems with an agar and collagen mixture. It seemed there was a synergistic effect between gelatin that formed during collagen hydrolysis and agar. The higher strength of the obtained gel matrix was also confirmed by texture analysis, as firmness values were almost two times higher (2.2–4.8 N) than in systems with agar only (1.1–2.25 N) (Figure 5). Firmness is one of the most important properties of structured oils and spreads [37].

**Figure 6:**
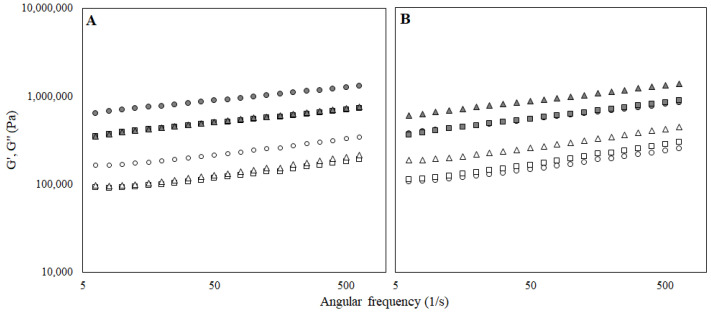
Elastic (G′) and loss modulus (G″) of the bigel-based vegetable oil spreads structured with agar (**A**) or agar and collagen mixture (**B**) with different concentrations of lecithin (○—0.5%; □—1.0% lecithin; ∆—1.5%) in the oleogel fraction.

With regard to the rheological properties, all samples revealed a typical gel network formation as the elastic modulus (G′) was always above the loss modulus (G″) and both moduli were separated by less than one logarithmic cycle (with the tan δ always below 1) (Figure 6). Such behaviour was previously reported as typical for gels and spreads [31,38,39]. Additionally, a slight frequency dependence was observed for both moduli, suggesting that although the gels were strong, they showed a slightly weak network. Evaluating the distance between the two modules, it can be assumed that the gels stabilized with gelatin were stronger. Moreover, there was no apparent connection between lecithin content and frequency dependence.

We had some asummptions that the different nature and stabilizing mechanism of agar could cause more spreadable and cohesive structure formation in comparison with gelatin. However, the obtained results showed that spreads had the same properties as with gelatin addition (similar stability, spreadability and visual appearance).

### 3.3. Oxidative Stability of Bigel-Based Vegetable Oil Spreads with Lingonberry Pomace, Structured with Gelatin or Agar

This section presents information on the oxidative stability in the bigel-based vegetable oil spreads with lecithin concentration of 0.5%, depending on the added hydrophilic structure-forming agent and the presence of the lingonberry pomace. At this stage of the experiment, we only chose samples with agar or gelatin, whereas samples with additional collagen were evaluated as being less acceptable because of their visual appearance.

**Figure 7:**
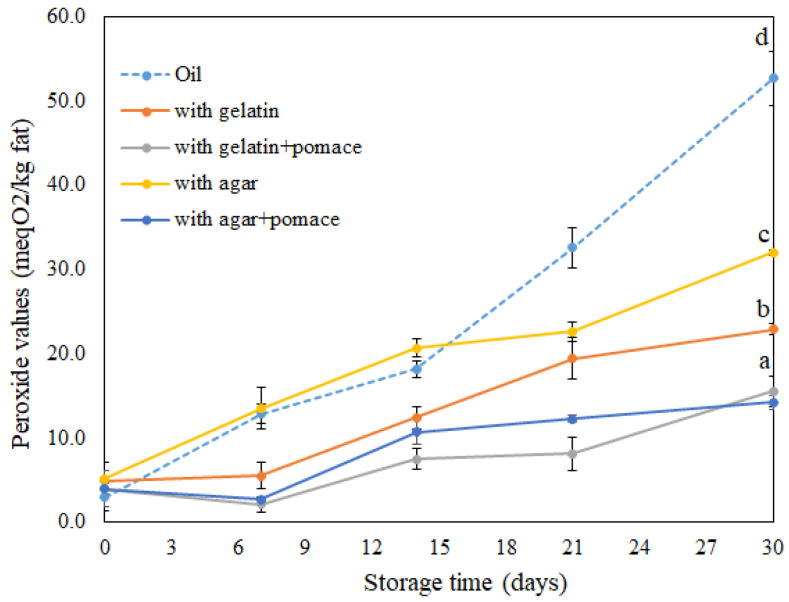
Peroxide values of different bigel-based vegetable oil spreads formulations (lecithin concentration in the oleogel phase–0.5%). Data labeled with different letters showed statistically significant differences (*p* < 0.05) between bigel-based vegetable oil spreads formulation.

The accumulation of oxidation products in all samples was found to have the expected exponential character (Figure 7). A high degree of oxidation development was obtained after seven days of storage in pure oil and bigel with agar, as PV values were above 10 mEq/kg [40]. The pure oil (control) showed the worst oxidative stability throughout the whole storage period, with a sharp increase in peroxide value from the 14th to the 30th day of storage (Figure 7). Lower oxidation development was recorded after 14 days of storage in bigel samples structured with gelatin and agar. These findings demonstrate that oil structuring helps to prevent oxygen contact with lipid droplets by having a positive effect on the resistance of the bigel samples to oxidation, which is consistent with other studies [41,42]. It was also obtained that higher peroxide values existed in the system with agar compared to those that contained gelatin. This could be related with the antioxidant capacity of gelatin that was also previously reported by other studies. Moreover, the antioxidant properties of gelatin mostly related to its hydrophobicity and amino acids composition [43].

Samples with pomace addition demonstrated the lowest level of primary oxidation product accumulation at the end of the study (14.22 ± 0.86 and 15.55 ± 1.82 mEq/kg). Such differences in the dynamics of peroxide accumulation are most likely related to the natural presence of phenolic compounds (total phenolic content-6.26 GAE/g d.m.) in the lingonberry pomace. The antioxidant activity of these compounds was previously reported by different authors [19]. Phenolic compounds are characterized by antioxidant activity due to a phenol ring which can provide hydrogen bonds from hydroxyl groups and delocalize unpaired electrons [44]. The obtained results were also confirmed by the antioxidant capacity of the bigel samples that was evaluated by DPPH^•^scavenging. As can be seen in Table 5, the presence of the lingonberry pomace enhanced the resistance of the bigel samples after preparation (1.61 ± 0.12; 2.03 ± 0.01 mg TE/g) and after 30 days of storage (1.14 ± 0.09; 1.43 ± 0.15 mg TE/g). The higher declining rate of DPPH inhibiting capacity in the samples with pomace addition (from 1.61 to 1.14 mg TE/g and from 2.03 to 1.42 mg TE/g) could be related to the degradation of phenolics during storage. The autooxidation and degradation of phenolics during storage under different conditions were previously reported in dried papayas and apricots [45,46].

The oxidative stability of the structured bigel-based vegetable oil spreads were also monitored by a pressure drop in the oxipress system. A prolonged oxidation induction period was also obtained in the systems with pomace addition, and such results are in good correlation with peroxide values and DPPH^•^ inhibition.

## 4. Conclusions

This study shows that it is possible to formulate agar or gelatin structured bigel-based vegetable oil spreads enriched with lingonberry pomace while maintaining the structural properties of traditional spreads. All formulated bigels were stable and characterized as easy spreadable. The addition of ligonberry pomace affected grainy structure formation with the homogenous and uniform distribution of fiber particles throughout the bigel matrix and it also altered the colour of the bigels toward a purple-red. It was also found that dynamic stability, rheological, textural properties as well as colour were affected by the type of the structuring agent and lecithin concentration.

The formulated bigel-based spreads could have a potential application as healthier fat spreads. Moreover, the added lingonberry pomace showed dual functionality: it acts as a source of the fiber and reduces the development of the oxidation process. All spreads could be characterized as high in fibre (11 g of fibre per 100 g of the spread) with high energetic values (564 to 598 kcal).

## Figures and Tables

**Table 1:** Composition (%) of bigel-based vegetable oil spreads structured with gelatin or gelatin and collagen.

	Structured with Gelatin			Structured with Gelatin and Collagen		
Carnauba wax	3.60			3.60		
Lecithin	0.3	0.6	0.9	0.3	0.6	0.9
Oil	56.10	55.80	55.50	56.10	55.80	55.50
Water	22.8			16.55		
Gelatin	2.0			2.0		
Collagen	-			6.25		
Lingonberry pomace	15.0			15.0		
Stevia sweetener	0.2			0.2		

**Table 2:** Composition (%) of bigel-based vegetable oil spreads structured with agar or agar and collagen.

	Structured with Agar			Structured with Agar and Collagen		
Carnauba wax	3.60			3.60		
Lecithin	0.3	0.6	0.9	0.3	0.6	0.9
Oil	56.10	55.80	55.50	56.10	55.80	55.50
Water	23.8			17.55		
Agar	1.0			1.0		
Collagen	-			6.25		
Lingonberry pomace	15.0			15.0		
Stevia sweetener	0.2			0.2		

**Table 3:** Colour characteristics and dynamic stability of bigel-based vegetable oil spreads structured with gelatin or with a gelatin and collagen mixture with different concentrations of lecithin (0.5, 1.0 and 1.5%) in the oleogel fraction.

	Structured with Gelatin			Structured with Gelatin and Collagen		
	Lecithin Amount in Oleogel Fraction,%					
	0.5	1.0	1.5	0.5	1.0	1.5
L*	31.49 ± 0.44 ^c^	29.68 ± 0.06 ^b^	28.28 ± 0.22 ^a^	27.71 ± 0.33 ^c^	25.01 ± 0.06 ^b^	24.35 ± 0.04 ^a^
a*	17.66 ± 0.40 ^a^	18.01 ± 0.15 ^a^	19.99 ± 0.25 ^b^	5.63 ± 0.08 ^a^	9.34 ± 0.12 ^b^	9.60 ± 0.06 ^c^
b*	3.42 ± 0.04 ^a^	3.62 ± 0.04 ^b^	4.52 ± 0.06 ^c^	0.56 ± 0.06 ^a^	1.13 ± 0.03 ^b^	1.44 ± 0.02 ^c^
TFR (%)	16.11 ± 1.29 ^a^	19.52 ± 2.16 ^b^	21.82 ± 1.52 ^c^	17.75 ± 0.87 ^a^	24.50 ± 2.95 ^c^	25.93 ± 1.88 ^c^
VR (%)	0.25 ± 0.03 ^a^	0.20 ± 0.02 ^a^	0.47 ± 0.02 ^b^	0.17 ± 0.02 ^b^	1.30 ± 0.01 ^c^	0.08 ± 0.0 ^a^
FR (%)	99.75 ± 1.32 ^ab^	99.80 ± 2.16 ^b^	99.45 ± 1.50 ^ab^	99.83 ± 0.88 ^ab^	98.69 ± 1.87 ^ab^	99.92 ± 1.89 ^b^

Values are presented as mean values ± standard deviation. Data labeled with different letters in rows showed statistically significant differences (*p* < 0.05) between bigel-based vegetable oil spreads formulation with different lecithin concentrations.

**Table 4:** Characteristics of bigel-based vegetable oil spreads structured with agar or an agar and collagen mixture with different concentrations of lecithin in the oleogel fraction.

	with Agar			with Agar and Collagen		
	Lecithin Amount in Oleogel Fraction,%					
	0.5	1.0	1.5	0.5	1.0	1.5
L*	27.19 ± 0.04 ^b^	29.59 ± 0.58 ^ab^	31.35 ± 0.35 ^ab^	25.64 ± 0.02 ^a^	26.21 ± 0.23 ^b^	26.17 ± 0.38 ^b^
a*	16.48 ± 0.15 ^b^	14.76 ± 0.40 ^a^	16.02 ± 0.22 ^b^	11.22 ± 0.03 ^b^	9.93 ± 0.02 ^a^	10.06 ± 0.13 ^a^
b*	3.06 ± 0.04 ^b^	2.60 ± 0.18 ^a^	3.00 ± 0.04 ^b^	1.54 ± 0.02 ^b^	1.38 ± 0.06 ^a^	1.51 ± 0.02 ^b^
TFR (%)	18.88 ± 0.47 ^a^	23.75 ± 0.45 ^b^	25.68 ± 1.67 ^c^	10.04 ± 1.90 ^a^	11.77 ± 2.05 ^b^	14.50 ± 1.55 ^c^
VR (%)	0.21 ± 0.03 ^a^	0.29 ± 0.02 ^b^	0.27 ± 0.01 ^b^	0.40 ± 0.02 ^a^	0.59 ± 0.01 ^b^	0.55 ± 0.02 ^b^
FR (%)	99.84 ± 0.44 ^b^	99.71 ± 0.41 ^ab^	99.73 ± 1.68 ^ab^	99.60 ± 1.90 ^ab^	99.83 ± 2.84 ^b^	99.45 ± 1.57 ^ab^

Values are presented as mean values ± standard deviation. Data labeled with different letters in rows show statistically significant differences (*p* < 0.05) between bigel-based vegetable oil spread formulations with different lecithin concentrations.

**Table 5:** Antioxidant efficiency characteristics of lingonberry pomace and DPPH^•^ inhibition of differently structured bigel-based vegetable oil spreads (lecithin concentration in the oleogel phase–0.5%) at 120 °C.

Bigel Sample	Fresh			After 30 Days		
	IP (h)	PF	DPPH^•^ Inhibition (mg TE/g)	IP (h)	PF	DPPH^•^ Inhibition (mg TE/g)
with gelatin	1.75 ± 0.10 ^a^	1.0	0.26 ± 0.01 ^a^	1.77 ± 0.07 ^b^	1.0	0.21 ± 0.06 ^a^
with gelatin + pomace	4.22 ± 0.01 ^b^	2.4	1.61 ± 0.12 ^b^	4.14 ± 0.01 ^d^	2.3	1.14 ± 0.09 ^b^
with agar	1.95 ± 0.11 ^a^	1.0	0.28 ± 0.02 ^a^	1.40 ± 0.02 ^a^	1.0	0.23 ± 0.01 ^a^
with agar + pomace	4.03 ± 0.12 ^b^	2.1	2.03 ± 0.01 ^c^	3.98 ± 0.03 ^c^	2.8	1.43 ± 0.15 ^c^

Values are presented as mean values ± standard deviation. Data labeled with different letters in columns showed statistically significant differences (*p* < 0.05) between bigel-based vegetable oil spread formulations.

## Data Availability

The data presented in this study are available on request from the corresponding author.
